# The Ubiquitin Ligase Ubr2, a Recognition E3 Component of the N-End Rule Pathway, Stabilizes Tex19.1 during Spermatogenesis

**DOI:** 10.1371/journal.pone.0014017

**Published:** 2010-11-16

**Authors:** Fang Yang, Yong Cheng, Jee Young An, Yong Tae Kwon, Sigrid Eckardt, N. Adrian Leu, K. John McLaughlin, Peijing Jeremy Wang

**Affiliations:** 1 Department of Animal Biology, Center for Animal Transgenesis and Germ Cell Research, University of Pennsylvania School of Veterinary Medicine, Philadelphia, Pennsylvania, United States of America; 2 Department of Pharmaceutical Sciences, Center for Pharmacogenetics, University of Pittsburgh, Pittsburgh, Pennsylvania, United States of America; 3 Department of Molecular Medicine and Biopharmaceutical Sciences, Graduate School of Convergence Science and Technology, Seoul National University, Seoul, Korea; 4 Research Institute at Nationwide Children's Hospital, Columbus, Ohio, United States of America; Temasek Life Sciences Laboratory, Singapore

## Abstract

Ubiquitin E3 ligases target their substrates for ubiquitination, leading to proteasome-mediated degradation or altered biochemical properties. The ubiquitin ligase Ubr2, a recognition E3 component of the N-end rule proteolytic pathway, recognizes proteins with N-terminal destabilizing residues and plays an important role in spermatogenesis. Tex19.1 (also known as Tex19) has been previously identified as a germ cell-specific protein in mouse testis. Here we report that Tex19.1 forms a stable protein complex with Ubr2 in mouse testes. The binding of Tex19.1 to Ubr2 is independent of the second position cysteine of Tex19.1, a putative target for arginylation by the N-end rule pathway R-transferase. The *Tex19.1*-null mouse mutant phenocopies the *Ubr2-*deficient mutant in three aspects: heterogeneity of spermatogenic defects, meiotic chromosomal asynapsis, and embryonic lethality preferentially affecting females. In *Ubr2*-deficient germ cells, *Tex19.1* is transcribed, but Tex19.1 protein is absent. Our results suggest that the binding of Ubr2 to Tex19.1 metabolically stabilizes Tex19.1 during spermatogenesis, revealing a new function for Ubr2 outside the conventional N-end rule pathway.

## Introduction

The N-end rule pathway is a ubiquitin-dependent proteolytic system [1], [2]. In this pathway, the stability of proteins is defined by their N-terminal amino acids that are distinguished into stabilizing and destabilizing residues. The latter constitute so-called N-degrons, which are signatures for degradation of short-lived proteins. Destabilizing residues include basic (Arg, Lys, and His) and bulky hydrophobic (Phe, Tyr, Trp, Leu, and Ile) residues. An N-degron can also be created by either endoproteolytic cleavage or modifications of a pre-N-degron (Cys, Asn, Asp, Gln, or Glu) through a series of N-terminal modifications [2]. Cysteine at position 2 (after methionine) is a unique type of destabilizing residue in mammalian cells. If N-terminally exposed, Cys can be oxidized to Cys-O_2_(H) or Cys-O_3_(H) before being arginylated by the arginine (R)-transferase ATE1 [3]–[5]. The N-degron is recognized by a family of UBR box-containing E3 ligases [6]. The mammalian genome encodes at least four UBR members (Ubr1, Ubr2, Ubr4 and Ubr5) characterized by the UBR box, a ∼70-residue zinc finger-like domain [2], [6].

N-end rule substrates known to contain N-degrons include a set of cardiovascular GPCR regulators (RGS4, RGS5, and RGS16) in mammals [3], [5], *D. melanogaster* DIAP1 [7], and *S. cerevisiae* cohesin component Scc1 [8]. Substrates targeted through internal degradation signals include histone H2A in mouse spermatocytes [9], *S. cerevisiae* Cup9 (a transcriptional repressor of the peptide transporter Ptr2) [10], and mammalian c-Fos [11].

Genetic studies have revealed that the N-end rule pathway plays an important role in many biological processes, including cardiac development, angiogenesis, and meiosis. *Ate1*-deficient mice die at fetal stages due to cardiovascular defects [4]. Mutations in the human *UBR1* gene cause Johanson-Blizzard syndrome, which is characterized by exocrine pancreatic insufficiency, multiple malformations and mental retardation [12]. Disruption of *Ubr2* in mice causes spermatogenic defects and female lethality [13]. Ubr2 localizes to meiotic chromatin regions and functions together with the ubiquitin conjugating (E2) enzyme HR6B in histone H2A ubiquitylation during male meiosis [9].

We previously identified *Tex19.1* (also known as *Tex19*) as a gene with germ cell-specific expression in the testis [14]. Disruption of *Tex19.1* causes defects in spermatogenesis [15]. Here we demonstrate that Tex19.1 forms a stable complex with Ubr2 during spermatogenesis. In *Ubr2*-deficient germ cells, Tex19.1 protein is absent despite abundant *Tex19.1* mRNA, suggesting that Ubr2 is required for stabilization rather than degradation of Tex19.1 during spermatogenesis.

## Results

### Tex19.1 forms a stable complex with Ubr2

**Figure 1:**
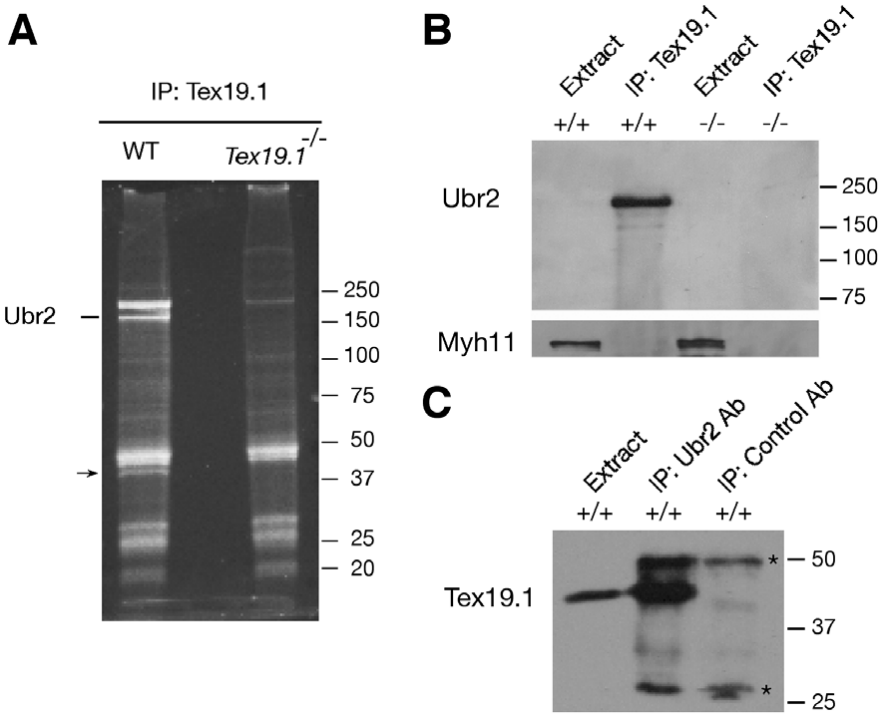
Tex19.1 interacts with Ubr2 in the testis. Testicular protein extracts prepared from 20-day-old wild type and *Tex19.1* ^−/−^ mice were used for co-immunoprecipitation (IP) experiments. (A) Identification of Tex19.1-associated proteins from testis by mass spectrometry. Tex19.1-associated proteins were co-immunoprecipitated from testicular extracts with affinity-purified antibody, and analyzed by SDS-PAGE and SYPRO Ruby staining. To confirm specificity, IP of proteins from *Tex19.1^−/−^* testes was performed in parallel. A second differentially expressed band (lower mass, indicated by arrow) was identified as actin by mass spectrometry. (B) Co-IP of Ubr2 with Tex19.1 from testis. IP was performed with the anti-Tex19.1 antibody and probed with the anti-Ubr2 antibody. Note that Ubr2 was too low in abundance in the total testicular extract to be detected by western blot analysis. Myh11 (myosin heavy chain 11) served as a loading control. (C) Reciprocal Co-IP experiment. Tex19.1 was co-immunoprecipitated with anti-Ubr2 but not control antibody. Bands indicated by asterisks in the IP are likely to be antibody light/heavy chains or non-specific species. Molecular mass standards are shown in kilodaltons.

Mouse Tex19.1 is a small protein (351 aa) with a coiled-coil domain, which is known to mediate protein-protein interactions [14]. To identify potential binding partners of Tex19.1, we performed immunoprecipitation (IP) experiments with testicular protein extracts using a Tex19.1-specific antibody that we generated. One prominent protein band (∼200 kD) was found in the co-immunoprecipitated proteins from wild type testes but not *Tex19.1* ^−/−^ testes (Fig. 1A). Mass spectrometry analysis identified this band as Ubr2, one of the recognition E3 components of the N-end rule pathway [13].

**Figure 2:**
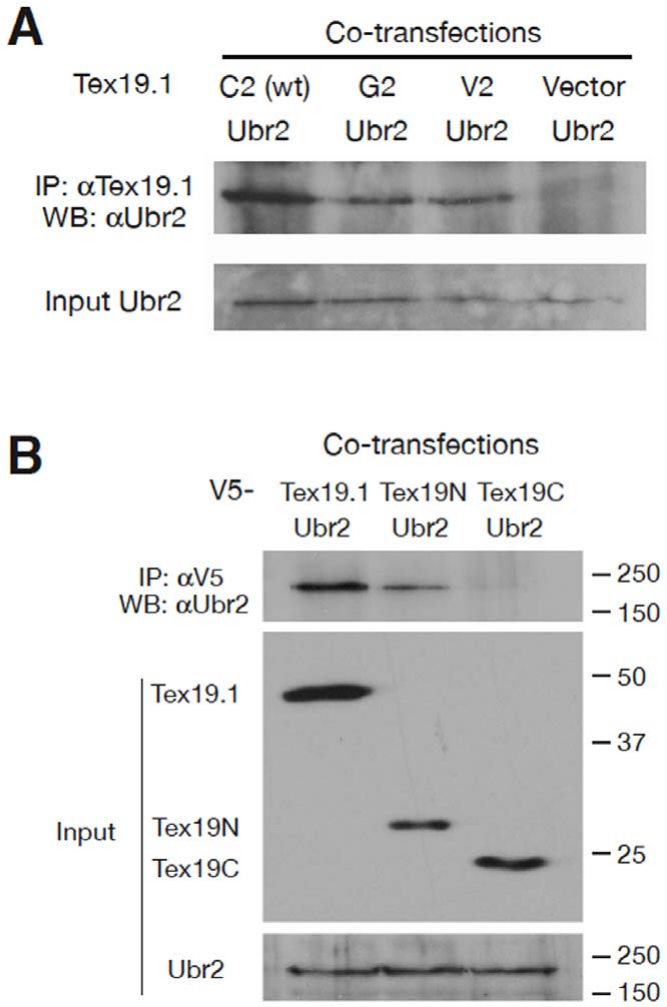
Binding of Ubr2 and Tex19.1 is arginylation-independent. All co-transfections were performed in NIH3T3 cells. (A) Co-immunoprecipitation of Ubr2 with C2-Tex19.1 (wild type), C2G-Tex19.1, and C2V-Tex19.1 demonstrates no difference in interaction between wild-type and mutant proteins. (B) Ubr2 interacts with the evolutionarily conserved N-terminal half of Tex19 but not with the C-terminal half. Mouse Tex19.1, 351 aa, pI = 4.69; Tex19.1N, aa 1–163, pI = 4.15; Tex19.1C, aa 164–351, pI = 6.34. All Tex19 proteins were tagged with V5 epitope at the N-termini. The slow migration of both full-length Tex19.1 and Tex19N on SDS-PAGE was due to their low pI. Molecular mass standards are shown in kilodaltons.

To verify the interaction between Tex19.1 and Ubr2, we performed co-immunoprecipitation experiments followed by Western blot analysis with specific antibodies. The abundance of Ubr2 in testis was too low to be detected in total testicular extract (Fig. 1B). However, Ubr2 was readily detectable in the protein fraction immunoprecipitated with the anti-Tex19.1 antibody from wild type but not from *Tex19.1*-deficient testes (Fig. 1B). Likewise, Tex19.1 was co-immunoprecipitated in the reciprocal IP with the anti-Ubr2 antibody (Fig. 1C), demonstrating that Tex19.1 and Ubr2 are associated with each other in the testis. Co-transfection of NIH3T3 cells followed by co-immunoprecipitation and Western blot analysis further support the interaction between Tex19.1 and Ubr2 (Fig. 2A, Lane 1).

### Interaction of Ubr2 with Tex19.1 is arginylation-independent

In the mammalian N-end rule pathway, Cys at position 2 (after Met) is a unique type of destabilizing residue. Cleavage of the first Met by Met aminopeptidase exposes Cys at position 2. Tex19.1 from all species examined bears an N-terminal Met^1^-Cys^2^ sequence, a putative pre-degron that can destabilize a substrate through oxidation and arginylation [3]–[5]. To test whether the binding of Tex19.1 by Ubr2 requires the N-terminal arginylation of Tex19.1 Cys^2^, we generated two different mutant Tex19.1 proteins in which cysteine (C2) was replaced with either glycine (G2) or valine (V2). Co-transfection and co-IP experiments using NIH3T3 cells showed that Ubr2 binds to both Tex19.1 mutant proteins, demonstrating that the interaction between Ubr2 and Tex19.1 does not require Cys^2^ (Fig. 2A, Lanes 2 and 3).

### Ubr2 binds to the evolutionarily conserved N-terminal region of Tex19.1

The human genome contains only one *TEX19* gene. Human TEX19 protein consists of only 164 aa and is homologous to the N-terminal part of mouse Tex19.1 protein (aa 1–162). This partial conservation suggests that the N-terminal half of Tex19 interacts with Ubr2. To test this hypothesis, we generated two partial murine Tex19.1 proteins consisting of either the N or the C-terminal half: Tex19.1N (aa 1–163) and Tex19.1C (aa 164–351). Co-IP analyses of these proteins with Ubr2 demonstrated that Ubr2 binds to the evolutionarily conserved N-terminal half but not the unconserved C-terminal half of Tex19 (Fig. 2B).

### Ubr2 interacts with Tex19.2

**Figure 3:**
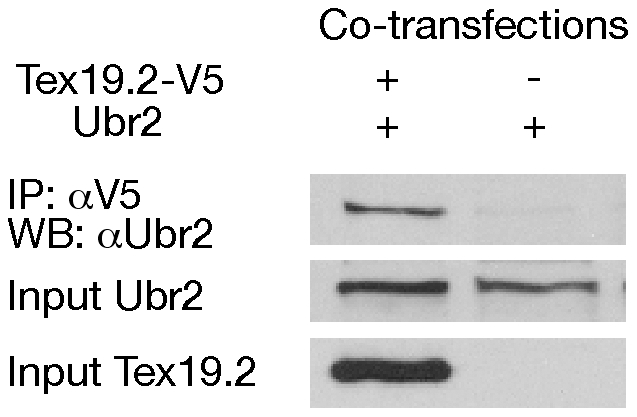
Ubr2 interacts with Tex19.2. Co-transfections were performed in NIH3T3 cells. Tex19.2 was tagged with the V5 epitope.

Murine *Tex19.1* forms a two-gene family with its sequence homologue *Tex19.2*, which is separated by only 27 kb from *Tex19.1*, but transcribed independently. While *Tex19.1* is expressed specifically in germ cells, *Tex19.2* expression is restricted to testicular somatic cells [16]. Using co-transfection and co-IP assays, we found that Ubr2 also binds to Tex19.2 (Fig. 3).

### *Tex19.1*-null mouse mutant phenocopies *Ubr2*-null mutant

**Figure 4:**
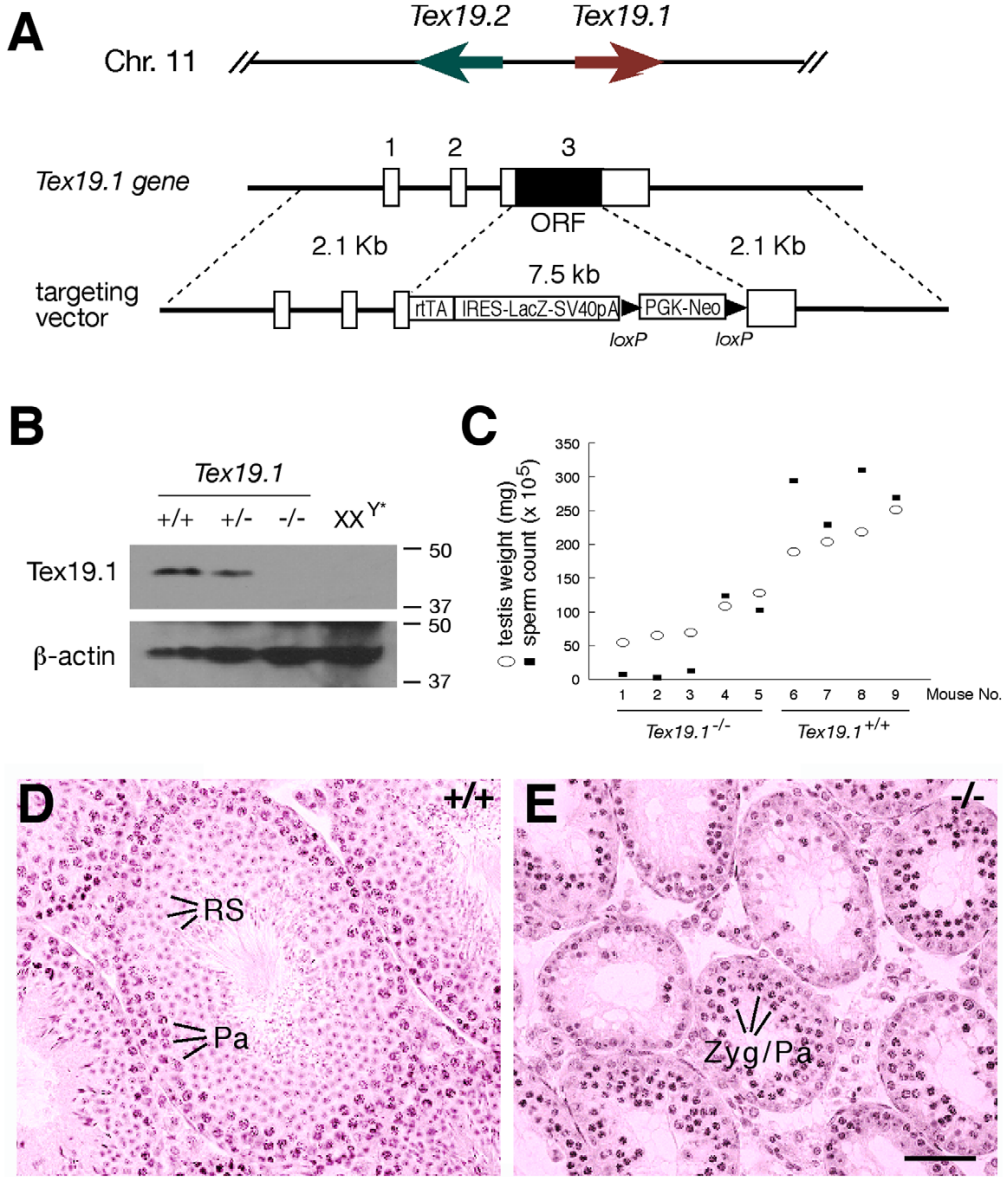
Meiotic defects in *Tex19.1* ^−/−^ males. (A) Schematic diagram of the *Tex19.1* targeting strategy. *Tex19.1* and *Tex19.2* are located only 27 kb apart but are transcribed in opposite orientations. All three exons of *Tex19.1* are drawn to scale as rectangles and are designated by numbers shown above. The entire *Tex19.1* ORF is located in the last exon and is replaced by the rtTA2^S^-M2-IRES-*LacZ*-PGK-Neo cassette in the mutant allele. PGK-Neo is flanked by *loxP* sites. The expression of rtTA (reverse tetracycline-controlled transactivator) and *lacZ* is expected to be driven by the *Tex19.1* promoter. (B) Absence of Tex19.1 protein in *Tex19.1* ^−/−^ testes. Western blot analysis was performed on 20 µg each of adult wild type, *Tex19.1* ^+/−^, *Tex19.1* ^−/−^, and XX^Y*^ testicular extracts. XX^Y*^ testes are depleted of germ cells and were used as controls. Molecular mass standards are shown in kilodaltons. (C) Sperm output correlates with testis weight. Five *Tex19.1* ^−/−^ and four wild type mice of 2–3 months of age were plotted for testis weight and epididymal sperm count. (D, E) Histological analysis of testes from 3-month-old wild type (D) and *Tex19.1* ^−/−^ (E) testes. Zyg/Pa, zygotene/pachytene spermatocytes; RS, round spermatids. Scale bar, 50 µm.

*Ubr2* ^−/−^ mice exhibit spermatogenic defects and embryonic lethality preferentially affecting females [13]. Because Tex19.1 and Ubr2 form a stable complex in the testis, we next investigated the consequence of loss of this interaction in the *Tex19.1*-null mice. We disrupted the *Tex19.1* gene by homologous recombination in embryonic stem (ES) cells (Fig. 4A). *Tex19.1* consists of three exons with the entire coding region (ORF) residing in the last exon. In the targeted allele, the ORF of *Tex19.1* was replaced by the selection marker (Neo), rtTA and *LacZ*. Western blot analysis confirmed the absence of the Tex19.1 protein in *Tex19.1* ^−/−^ testes (Fig. 4B). The testes of sterile XX^Y*^ mice completely lack germ cells. The absence of Tex19.1 in XX^Y*^ testes (Fig. 4B) demonstrates that Tex19.1 is germ cell-specific in the testis, consistent with previous studies [14]–[17]. In contrast with *Tex19.1*, *Tex19.2* is expressed in somatic cells of the testis [16]. Therefore, our Western blot data (Fig. 4B) also shows that our antibody is specific to Tex19.1 and does not recognize Tex19.2, due to limited amino acid sequence homology (58% identity) between Tex19.1 and Tex19.2.

No gross somatic defects were observed in adult *Tex19.1* ^−/−^ mice. However, interbreeding of heterozygous mice yielded a sub-mendelian ratio of genotypes of offspring (+/+: +/−: −/−; males: 61∶103∶35; females: 43∶84∶8). Significantly fewer homozygous mice were produced than expected (χ^2^ = 27.07, two degrees of freedom, p<0.0001), suggesting embryonic lethality. In addition, the embryonic lethality preferentially affected females (χ^2^ = 26.22, two degrees of freedom, p<0.0001). As Tex19.1 protein is expressed in embryonic stem cells at substantial levels (detectable by Western blot analysis, data not shown), the observed embryonic lethality of *Tex19.1*-null mutant mice could be associated with consequences of lack of expression of *Tex19.1* in pluripotent stem cells during embryogenesis [16].

Disruption of *Tex19.1* resulted in sharply reduced testis size. The weight of *Tex19.1* ^−/−^ testes (104±48 mg/pair, n = 6 mice, p<0.001) from 2–3 month old mice was half (50%) that of *Tex19.1* ^+/−^ testes (210±34 mg/pair). Notably, the testes from *Tex19.1* ^−/−^ males varied dramatically in size (Fig. 4C). Likewise, the sperm output in the cauda epididymus from *Tex19.1* ^−/−^ mice varied greatly, ranging from 3.0×10^5^ to 1.2×10^7^, but correlated well with the testis size. In most *Tex19.1*-deficient testes, spermatogenesis appeared to be blocked at the meiotic stage with abundant zygotene or early pachytene-like spermatocytes but few postmeiotic germ cells (Fig. 4E). However, the severity of meiotic defects varied greatly from mouse to mouse, and even from tubule to tubule in the same testis. The most severe meiotic defect was meiotic arrest at early pachytene stage. In testes with less severe defects, round and elongating spermatids were present but at a greatly reduced number (data not shown). It is intriguing but unclear why the *Tex19.1*-deficient mice exhibit such a large degree of phenotypic variation.

We next examined whether *Tex19.1*-deficient spermatocytes exhibit defects in chromosomal synapsis by immunostaining of SYCP1 (transverse filaments) and SYCP2 (lateral elements) [18]–[20]. The axial elements of the synaptonemal complex were assembled in *Tex19.1*-deficient spermatocytes. 48% of *Tex19.1*-deficient pachytene spermatocytes had normal synapsis (Fig. S1A). In the remaining pachytene spermatocytes, we observed variable synaptic failure; some chromosomes were completely asynapsed, whereas the majority of chromosomes were fully synapsed (Fig. S1B–D). In contrast, only 3% of wild type pachytene nuclei (280 scored) had asynapsed chromosomes, which were all sex chromosomes. Specifically, of the *Tex19.1*-deficient pachytene spermatocytes, 16% contained only asynapsed sex chromosomes, 4% contained only one pair of asynapsed autosomes, in 27% 2–5 pairs of chromosomes were asynapsed and in 5% more than 5 pairs of chromosomes were affected (Fig. S1E). In conclusion, these data demonstrate that disruption of *Tex19.1* causes defects in chromosomal synapsis.

In summary, *Tex19.1* ^−/−^ mouse mutant that we generated confirms the spermatogenic phenotypes of a previously reported *Tex19.1* mutant [15]. In addition, our study has uncovered a novel finding that the embryonic lethality in *Tex19.1*-null mice preferentially affects females. Collectively, the *Tex19.1* ^−/−^ mouse mutant exhibits abnormal spermatogenesis, defective chromosomal synapsis, and incomplete penetrance of embryonic lethality preferentially affecting females, all of which are present in the *Ubr2* ^−/−^ mouse mutant [13]. The heterogeneity of spermatogenic defects was also observed in *Ubr2*-deficient mice. The testis weight of *Ubr2*-deficient testis ranged from 30 to 70% of the wild type controls and some *Ubr2*-deficient males produced sperm [13]. Thus, consistent with the interaction between Tex19.1 and Ubr2, these two mouse null mutants phenocopy each other.

### Depletion of the Tex19.1 protein in Ubr2-deficient testis

**Figure 5:**
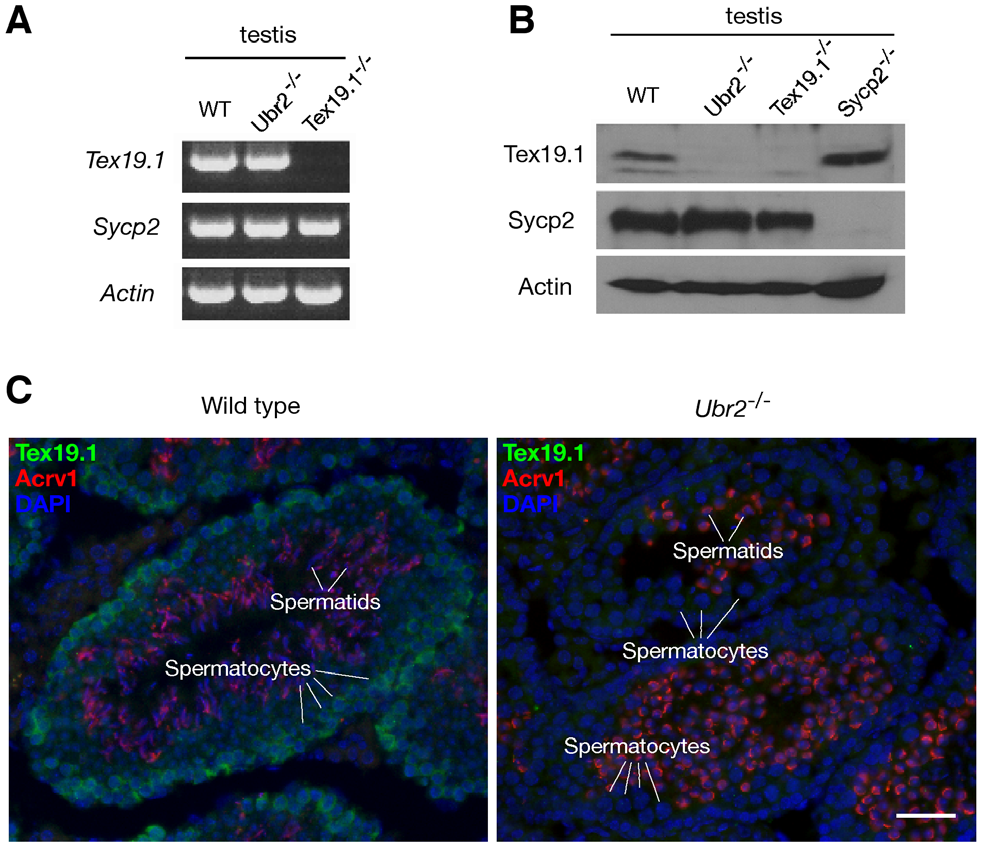
Depletion of the Tex19.1 protein in *Ubr2*-deficient testes. (A) Detection of *Tex19.1* transcript in *Ubr2* ^−/−^ testis by RT-PCR. *Sycp2* and *β-actin* served as germ cell-specific and ubiquitous expression controls [20]. (B) Western blot analysis shows the absence of Tex19.1 protein in *Ubr2* ^−/−^ testis. *Sycp2* ^−/−^ testis exhibits meiotic arrest and serves as a control [20]. (C) Loss of Tex19.1 protein in *Ubr2* ^−/−^ germ cells. Testis sections from adult wild type and *Ubr2* ^−/−^ mice were immunostained with anti-Tex19.1 and anti-Acrv1 antibodies. Acrv1 is a component of acrosomes and thus is only present in the haploid germ cells - spermatids. Note the abundance of round spermatids in the *Ubr2*-deficient testis. Scale bar, 25 µm.

Ubr2 is an ubiquitin E3 ligase of the N-end rule pathway [13]. If Tex19.1 were an in vivo substrate of Ubr2 and thus targeted for degradation, one would expect increased abundance of Tex19.1 protein in *Ubr2*-deficient testes. To test this possibility, we first analyzed *Tex19.1* transcript abundance in *Ubr2* ^−/−^ and wild type testes and found comparable levels (Fig. 5A). However, Tex19.1 protein was not detected in *Ubr2* ^−/−^ testes by Western blot analysis (Fig. 5B). The absence of Tex19.1 in *Ubr2* ^−/−^ testes was not due to loss of germ cells, since Sycp2, another meiosis-specific protein, was present in *Ubr2* ^−/−^ testes. Furthermore, we detected abundant Tex19.1 in *Sycp2* ^−/−^ testes exhibiting meiotic arrest (Fig. 5B) [20]. We then performed immunofluorescence analysis of testis sections to investigate the localization of Tex19.1 protein throughout meiosis in wild type compared to *Ubr2*-deficient germ cells. In early wild type germ cells, including meiotic spermatocytes, we observed that Tex19.1 localizes to the cytoplasm but not the nucleus. In contrast, Tex19.1 was not detected in post-meiotic spermatids, which express Acrv1, a component of the acrosomes (Fig. 5C) [21]. Consistent with our Western blot data, Tex19.1 protein was clearly absent from *Ubr2*-deficient germ cells at all stages (Fig. 5C). Therefore, the absence of Tex19.1 may be a major causative mechanism underlying spermatogenic defects in *Ubr2*-deficient mice. These results suggest that Ubr2 causes stabilization rather than degradation of the Tex19.1 protein in testes.

## Discussion

Ubr2 is the recognition E3 component of the N-end rule pathway, in which an ubiquitin ligase recognizes a destabilizing N-terminal residue as an essential component of N-degron. It has been shown that Ubr2 plays a role in transcriptional silencing of meiotic chromosomes through ubiquitination of histone H2A [9], [13]. Here, we provide several lines of evidence that Ubr2 plays a novel function outside the N-end rule pathway: protein stabilization. Firstly, we demonstrate that Ubr2 forms a stable complex with Tex19.1 in testis. If Tex19.1 were an enzymatic substrate of Ubr2 and thus destined for ubiquitin-dependent degradation, its association with Ubr2 might be too transient to be detected by co-IP. In fact, although Ubr2 protein was not detectable in the total testicular extracts by Western blot, it was readily co-immunoprecipitated with Tex19.1, suggesting the formation of a stable protein complex. Secondly, Tex19.1 bears an N-terminal cysteine. Therefore, Tex19.1 might be a substrate of ATE1-dependent arginylation and Ubr2-dependent ubiquitylation. However, our results show that the Ubr2-Tex19.1 interaction does not require the evolutionarily conserved Cys^2^ residue, a putative arginylation substrate. Thirdly, the *Tex19.1* mouse mutant phenocopies the *Ubr2* null mutant, underscoring the physiological relevance of the Tex19.1-Ubr2 interaction. Lastly, the Tex19.1 protein is absent rather than more abundant in *Ubr2*-deficient testes. As Ubr2 binds to Tex19.1, the most parsimonious explanation is that Ubr2 stabilizes Tex19.1 metabolically. However, we cannot exclude the possibility that Ubr2 might also play a role in the translation of the *Tex19.1* mRNA.

The notion that Ubr2 plays a non-canonical function in protein turnover is further supported by the study of RECQL4 [22]. RECQL4, a putative DNA helicase, is mutated in the Rothmund–Thomson and RAPADILINO syndromes [23], [24]. Although RECQL4 forms a stable complex with both Ubr1 and Ubr2, RECQL4 is not ubiquitylated and is a long-lived protein in Hela cells, suggesting that it might also be stabilized by Ubr1/Ubr2 under certain conditions [22]. In our study, Ubr2 is required for the stability of Tex19.1. However, it is unclear how Ubr2 prevents Tex19.1 from degradation. One possibility is that the stable binding of Ubr2 to Tex19.1 blocks the accessibility of Tex19.1 by other E3 ligases.

## Materials and Methods

### Generation of anti-Tex19.1 and anti-Ubr2 polyclonal antibody

The entire mouse *Tex19.1* coding region was cloned into the pQE30 vector (QIAGEN). The 6xHis-Tex19.1 fusion protein was expressed in M15 bacteria, affinity-purified with Ni-NTA beads, and eluted in 8 M urea according to the manufacture's standard purification protocol (QIAGEN). The N-terminal 100 aa of mouse Ubr2 was expressed as a GST fusion protein in *E. coli* using the pGEX4T-1 vector and affinity purified with glutathione Sepharose. Each of the recombinant proteins was used to immunize two rabbits according to the company's standard protocol (Cocalico Biologicals, Inc). The anti-Tex19.1 antiserum (serum 2109) was used for western blot (1∶500). The anti-Ubr2 antiserum (serum 2186) was used for western blot (1∶500).

### Co-immunoprecipitation (IP), mass spectrometry and western blot analyses

To identify proteins coimmunoprecipitated with Tex19.1, testicular protein extracts were prepared from postnatal day 20 testes by homogenization in lysis buffer (50 mM HEPES, pH7.5, 140 mM NaCl, 1 mM EDTA, 10% glycerol, 0.5% NP-40, 1 mM PMSF). After centrifugation, S100 extracts were precleared by incubating with protein A agarose beads (Invitrogen) and centrifuged prior to use for IP with affinity-purified anti-Tex19.1 antibody. Immunoprecipitated proteins were washed four times in washing buffer (50 mM Tris HCl, pH 7.5, 250 mM NaCl, 0.1% NP-40, 0.05% deoxycholate) and were subject to 4–15% gradient SDS-PAGE analysis. The gel was stained with SYPRO Ruby (Bio-rad). Gel bands of interest were cut and sent to the Proteomics Core Facility at the University of Pennsylvania for protein identification by mass spectrometry. The following primary antibodies were used for co-IP and western blot analyses: anti-Ubr2 (Cat# H00023304-A01, Novus Biologicals; 1∶500), anti-Myh11 (Cat# ab683, Abcam; 1∶1000) and anti-β-actin (Cat# A5441, Sigma-Aldrich; 1∶5000). HRP-conjugated anti-rabbit and anti-mouse secondary antibodies (Sigma) were used.

### DNA constructs and transfection of cultured cells

The ORFs of mouse *Tex19.1*, *Tex19.2*, and *Ubr2* were cloned into pcDNA3.1/V5-His-TOPO mammalian expression vectors (Invitrogen) respectively. The V5 epitope tag was in frame only for Tex19.1, Tex19.2, Tex19.1N, and Tex19.1C (Fig. 2). Ubr2 was not tagged with the V5 epitope. Tex19.1 point mutations (C2G and C2V) were introduced through targeted mutation using PCR primers. All DNA constructs were verified by sequencing. DNA constructs were transfected into NIH3T3 cells (ATCC catalogue No. CRL-1658) using FuGENE6 Transfection Reagent (Invitrogen). IP and western blot analyses were performed with anti-V5 (Cat# 46-0705, Invitrogen) (1∶5000), anti-Tex19.1, and anti-Ubr2 antibodies.

### Targeted inactivation of the *Tex19.1* gene

In the *Tex19.1* targeting construct, two homologous arms (2.1 kb each) were amplified from a *Tex19.1*-positive BAC clone (RP23-400P17) by high-fidelity PCR and were subcloned to flank the rtTA2S-M2-IRES-LacZ-PGK-Neo knockin/selection cassette (Fig. 4). The rtTA2S-M2 fragment was PCR amplified from the pUHDrtTA2S-M2 plasmid [25]. Hybrid V6.5 XY ES cells (C57BL/6×129/sv) were electroporated with the linearized *Tex19.1* targeting construct (pUP77-29A/*Not*I) and were cultured in the presence of G418 (350 µg/ml). Electroporation was performed in a 0.4-cm Bio-Rad cuvette with the Bio-Rad Gene Pulser Xcell unit (Voltage, 400 v; Capacitance, 25 µF; Resistance, infinite; Expected time constant, 0.4 msec). Seven days after electroporation, 384 G418-resistant ES cell clones were picked. Screening of 96 clones by PCR identified twelve ES cell clones in which homologous recombination had occurred. PCR was performed with a forward primer upstream of the left arm and a reverse primer in the knockin/Neo cassette or a forward primer in the knockin/Neo cassette and a reverse primer downstream of the right arm. One targeted ES cell clone (3H6) was injected into B6C3F1 (Taconic) blastocysts that were subsequently transferred to the uteri of pseudopregnant ICR females. Male chimeras were bred with C57BL/6J females and germ-line transmission of the *Tex19.1* knockout/knockin allele was obtained. Mice from mixed strain backgrounds (C57BL/6×129/sv) were used in this study. In the *Tex19.1* mutant mice, the expression of rtTA and LacZ is expected to be under the control of the endogenous *Tex19.1* promoter. Offspring were genotyped by PCR of tail genomic DNA with the following primers: wild type (311 bp), ATGGATCCTGTCCCCCAGTCAGCGTT and GCGTCGACTTAGCACATAAAGGGACCCCAAT; mutant (450 bp), AAGTCGACATGTCTAGACTGGACAAGAG and CCTCCAATACGCAGCCCAGTGTAA. ∼3 mm-long tail biopsy was digested in 200 µl proteinase K buffer (10 mM Tris, 10 mM EDTA, 10 mM NaCl, 0.2% SDS, pH 8.0) at 55°C overnight. Genomic DNA was precipitated with 200 µl iso-propanol, washed once with 1 ml of 70% ethanol, air died and resuspended in 200 µl 1xTE buffer. 1 µl of genomic DNA was used in a 20 µl PCR genotyping reaction (94°C, 30 seconds; 55°C, 30 seconds; 72°C, 45 seconds; 35 cycles). Full details of the study were approved by the Institutional Animal Care and Use Committee (IACUC protocol # 802780) of the University of Pennsylvania.

### Histology, Surface spread, and immunofluorescent analyses

For histology, testes were fixed in Bouin's solution, embedded in paraffin, sectioned using the Leica RM2035 microtome, and stained with hematoxylin and eosin. Surface spread analyses of spermatocytes were performed as previous described [26]. For immunofluorescent analyses, adult wild type and *Ubr2* ^−/−^ testes were fixed with 4% paraformaldehyde overnight, dehydrated in 30% (w/v) sucrose solution, embedded with TBS tissue freezing medium (Fisher Scientific), and frozen at temperatures below −20°C. Sections (8 µm) were cut using a Reichert-Jung cryo-microtome. Sections were immunostained with anti-Tex19.1 and anti-Acrv1 antibodies [21]. For immunostaining of testis sections, specific anti-Tex19.1 antibodies were affinity-purified using the immunoblot method [27]. Texas Red and FITC-conjugated secondary antibodies were used.

## Supporting Information

Figure S1 TEX19 promotes chromosome synapsis. Spread nuclei of spermatocytes from 20-day-old Tex19-deficient mice were immunostained with anti-SYCP1 (green) and anti-SYCP2 (red) antibodies [19], [21]. (A) Tex19-deficient pachynema with apparently normal synapsis. Note the 19 pairs of fully synapsed autosomal homologues (yellow) and partially synapsed X-Y chromosomes (red). (B) Tex19-deficient pachynema with asynapsed XY only. Even though X and Y occupy the same nuclear domain, they remain separated. (C) Tex19-deficient pachynema with asynapsed XY and one pair of asynapsed autosomes (indicated by arrows). (D) Tex19-deficient pachynema with four pairs of asynapsed homologous chromosomes (red). (E) Analysis of asynapsed chromosomes in Tex19-deficient pachytene spermatocytes. ∼100 pachytene spermatocytes from each Tex19−/− mouse were examined for synapsis and divided into the five categories shown. Four 20-day-old Tex19−/− mice were analyzed. Values shown represent the mean ± standard deviation.
(0.71 MB TIF)
