# Supplementary figures and images for: The Ubiquitin Ligase Ubr2, a Recognition E3 Component of the N-End Rule Pathway, Stabilizes Tex19.1 during Spermatogenesis
Source: PLoS One. 2010 Nov 16;5(11):e14017. doi: 10.1371/journal.pone.0014017 (PMC2982839; doi:10.1371/journal.pone.0014017)

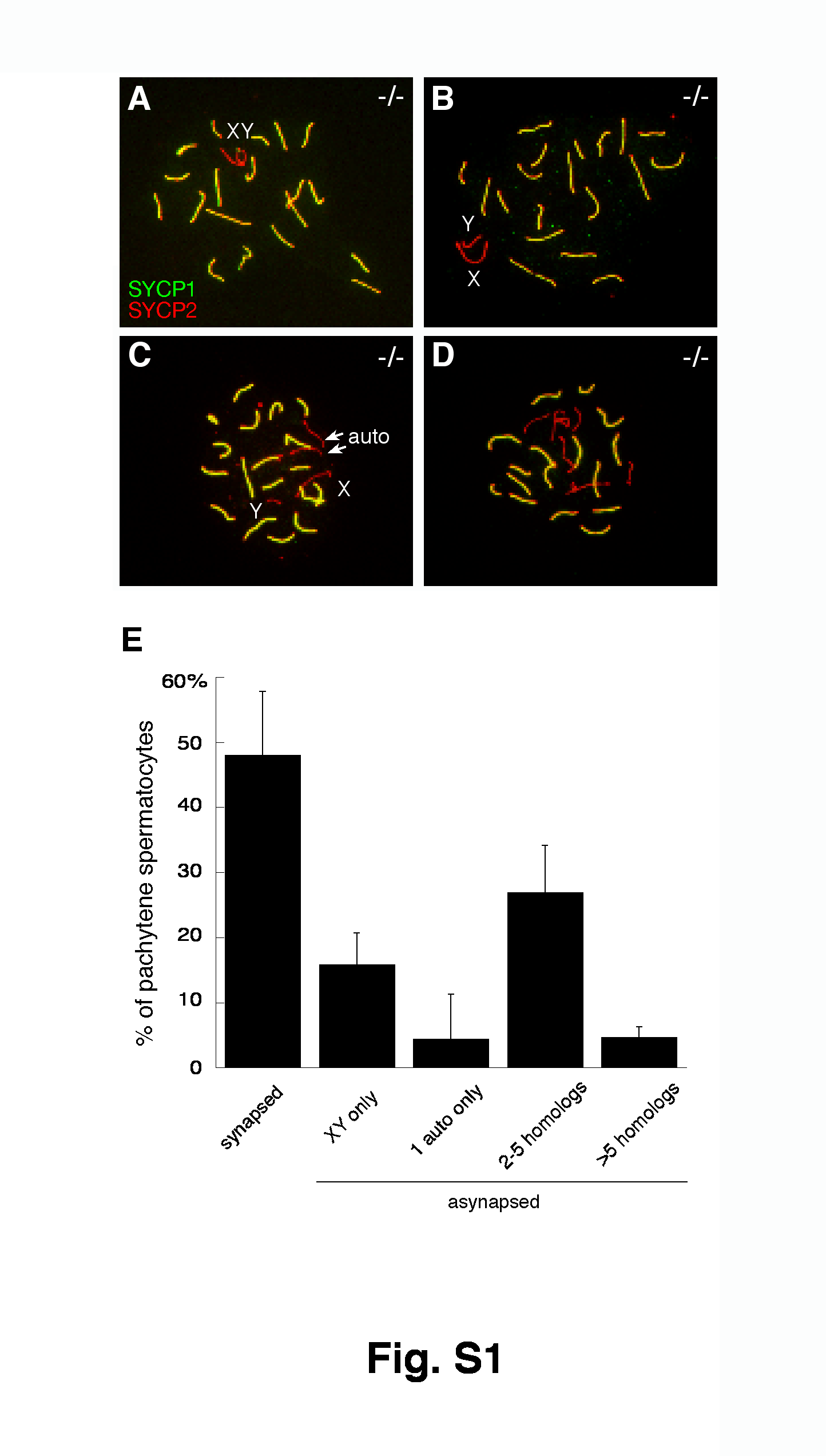

Supplement: Figure S1 — TEX19 promotes chromosome synapsis. Spread nuclei of spermatocytes from 20-day-old Tex19-deficient mice were immunostained with anti-SYCP1 (green) and anti-SYCP2 (red) antibodies [19], [21]. (A) Tex19-deficient pachynema with apparently normal synapsis. Note the 19 pairs of fully synapsed autosomal homologues (yellow) and partially synapsed X-Y chromosomes (red). (B) Tex19-deficient pachynema with asynapsed XY only. Even though X and Y occupy the same nuclear domain, they remain separated. (C) Tex19-deficient pachynema with asynapsed XY and one pair of asynapsed autosomes (indicated by arrows). (D) Tex19-deficient pachynema with four pairs of asynapsed homologous chromosomes (red). (E) Analysis of asynapsed chromosomes in Tex19-deficient pachytene spermatocytes. ∼100 pachytene spermatocytes from each Tex19−/− mouse were examined for synapsis and divided into the five categories shown. Four 20-day-old Tex19−/− mice were analyzed. Values shown represent the mean ± standard deviation. (0.71 MB TIF) [file pone.0014017.s001.tif]
